# Our daily helplessness: its presence and experience in the outpatient operation room of the Assisted Reproduction Clinic

**DOI:** 10.5935/1518-0557.20240082

**Published:** 2024

**Authors:** Márcia Christina Gonçalves Gusmão, Roberto de Azevedo Antunes, Marcelo Marinho de Souza, Ana Cristina Allemand Mancebo, Brunna Stumpo Vaz, Flávio Faria de Freitas, Maria do Carmo Borges de Souza

**Affiliations:** 1 Fertipraxis - Human Reproduction Center, Rio de Janeiro, RJ, Brazil

**Keywords:** psychotherapy, helplessness, operating room, assisted reproduction, IVF/ICSI, oocyte cryopreservation

## Abstract

**Objective:**

To identify the feeling of helplessness in assisted re production patients, along with the experience in the out patient surgical center of an assisted reproduction clinic.

**Methods:**

A prospective study of care and psychological interventions performed in the outpatient surgical center (OSC) of the assisted reproduction clinic from January 2019 to December 2022. Patients are first seen by the nursing staff. After an interview with the anesthesiologist and the attending physician, the psychotherapist presents herself and asks consent for listening/speaking, before, during and after the procedure.

**Results:**

1011 interviews were performed by the psychotherapist, which correspond to 47% of 2149 OSC procedures performed in the clinic during the study period. The psychotherapist was present in 595 IVF/ICSI (60%) of 1,000 procedures and 110 from 396 oocyte cryopreservation (28%), 306 (41%) from 753 transfers. The patients’ observations were written in their medical records. Relevant points were shared and discussed with the staff directly.

**Conclusions:**

The patients’ speeches addressed to the psychotherapist or to the multidisciplinary team in this environment contains the utterance of their feelings, conscious and unconscious, that affect their psyche. So, the feeling of helplessness, expressed and enunciated in the statements and conducts of patients as well as the team, may go unnoticed and not receiving the necessary care. In the OSC environment they are confronted with the reality that they would so much like to avoid, that is, to use the AR technique to achieve an unconsummated desired- pregnancy.

## INTRODUCTION

Many studies and articles have already dealt with the frustrations, anxieties, losses and anguish experienced by patients who look for the assisted reproduction clinic to form their families (Braverman *et al*., 2024; Golombok, 1992; Rooney & Domar, 2018). But, what about helplessness? At what moment can it be perceived, felt and heard in the ART clinic? Clinical practice has shown that listening to the experience and the feeling of helplessness has sometimes been left behind or even unnoticed.

Reflect on the experience of helplessness is important because “it makes it possible to think about its developments in the processes of subjectivation, making it possible to analyze the form of psychic organization of the subjects and the possible vicissitudes for helplessness” (Passos *et al*., 2018).

We can see that the notion of helplessness appears at different times in Freud’s writings, as well as in several articles and texts by contemporary psychotherapists and other theoretical approaches.

As this notion is fundamental for the constitution and understanding of the human psyche, we consider it relevant to know and understand its implications in the speeches, experiences of patients who enter the Outpatient Surgical Center (OSC) in the assisted reproduction clinic.

The term helplessness, according to Pereira (1999), should be considered more as a notion, and not as a well-defined concept in Freudian work. He mentions that Freud did not make a specific study on the subject. However, the non-formalization of a concept about this term would not remove its importance and relevance, considering that Freud made references to helplessness throughout his theoretical path, considering it vital for the constitution of the human psyche.

Patients desiring to have a child and build a Family may be faced with a feeling of helplessness since the beginning of ART. Although this search can provide a desired and satisfactory result, it does not, by itself, avoid an unscathed position for the patient. When faced with injectable medications and procedures (IVF/ICSI, oocyte cryopreservation, embryonic transfer) that will be performed at the OSC, they will be faced with their weaknesses. These may reactivate unique conditions of their state of helplessness, since the “requirement of the external world refers to the internal, to the psyche, emphasizing human vulnerability as a point that refers to helplessness” (Kislanov, 2002) (p. 6).

We used psychoanalytical theory and technique as a basis for understanding the notion of helplessness and its application in listening to patients who enter the AR clinic to undergo the procedures performed at the OSC. We consider as a reference for the context of helplessness, texts of Freudian metapsychology, such as: *Project for a scientific psychology* (Freud, 1977), *Inhibition, symptom and anguish* (Freud, 1926), *The future of an illusion* (Freud,1974a), *Civilization and its Discontents* (Freud,1974b); and contributions from other contemporary psychotherapists.

Building a family, having a child, is the desire and project of many couples and individuals. Since they fail to generate naturally, ART emerges as a possibility to achieve this desire and objective. Expectations, hopes are then created (terms that have an etymological relationship with the word hope) and hope that the child-desire will be fulfilled and the uterus filled. However, they do not imagine that along the way, or even during the procedures, they face frustrations, dissatisfactions and a feeling of helplessness.

The psychotherapist Suy (2022) considers that “our experiences with life always leave an unsatisfying remainder, a tone less satisfaction than we would like” (p. 70). On a daily basis, in the unbridled search for satisfaction caused by internal demands and the idealized contemporary media world, from which few escape, it is necessary to listen to “our daily helplessness”, including in the relationships established in the ART clinic.

## OBJECTIVE

To identify the feeling of helplessness in assisted reproduction patients, along with the experience in the outpatient surgical center of an assisted reproduction clinic.

## MATERIALS AND METHODS

The OSC resting room was identified in our group as a space for listening and welcoming the demands of patients (Gusmão *et al*., 2020). Based on this experience, the psychological care and follow-ups performed by the same psychotherapist at the Outpatient Surgical Center (OSC) of the Fertipraxis clinic, the patients were prospectively studied, from January 2019 to December 2022, during the procedures of oocyte collection, whether for IVF/ICSI, cryopreservation of oocytes, or for embryonic transfers. Fertipraxis Centro de Reprodução Humana is a private clinic located in Barra da Tijuca and Ipanema, Rio de Janeiro, Brazil.

All the patients, since beginning their medical care, whichever the indication of the procedure, receive detailed information about the stages of treatment, including the presence, performance and follow-up performed by the psychotherapist to the patients in attendance and/or in the OSC; they also receive contact numbers for the three directors and the nursing manager, to solve any doubts. Furthermore, there is clear information that any of the team’s physicians can be asked to clarify or resolve doubts during ultrasound control.

The clinical staff is made up of specialist physicians who perform attendance, ultrasound monitoring and are part of the procedures within the ASC. During the journey inside the clinic, a multidisciplinary team works: doctors, the therapist/psychologist, the nursing manager, nursing technicians, four embryologists and the pharmacist. In addition to its own patients, the clinic receives patients from associated physicians who use the OSC and ART laboratories. During this journey, meetings and clinical discussions are held for the purpose of uniformity of conduct and the adoption of continuing education measures.

All patients sign an informative consent form. The OSC complies with Brazilian operating regulations, according to the recent update brought through the Technical Standard on good practices in human cells, tissues and embryos, published on 12/26/2022 (Brazil, Ministério da Saude, ANVISA, 2022). The clinic has an operating license from ANVISA (National Surveillance Agency) and is Accredited by the Latin American Network of Assisted Reproduction (REDLARA).

The care performed by the psychotherapist at the OSC takes place on the days of the IVF/ICSI procedures, oocyte cryopreservation and embryo transfers, according to the schedule of the professional at the clinic. Each observation is recorded after in an electronic medical record and shared with the team, directly, when necessary.

The patient is initially assisted by the nursing team, which checks the vital signs, confirms the medications and previous tests performed. After contacting the anesthesiologist and the doctor who will perform the procedure, the psychotherapist introduces herself and asks for permission to have moments of listening/speaking, before, during and after the procedure. All attendance and data collected are recorded in an electronic medical record and shared with the team, directly, when necessary.

## RESULTS

A total of 1,011 patients were seen and monitored by the psychotherapist at the clinic’s OSC, from January 2019 to December 2022, when it was possible to identify situations in which the feeling of helplessness became evident. The psychological interventions carried out at the ASC, in a number of 1,011, corresponded to 47% of the total of 2,149 attendances in this period. At the time of oocyte aspiration in IVF/ICSI procedures, the presence of the professional enabled her to listen to 60% of the patients (595 cases attended), in relation to the total of 1,000 performed during this period. In the oocyte aspiration for cryopreservation, the percentage was 28% (110 cases), in a total of 396 procedures. Embryo transfers had a total of 753 undergoing psychological assistance 41% of the time (306 cases).

**Figure 1:**
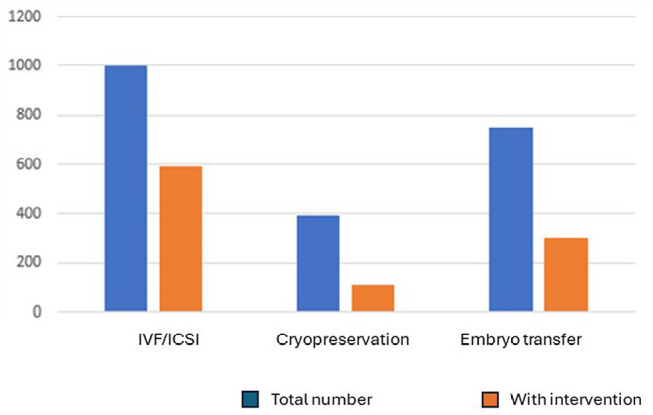
Procedures in the OSC without or with the intervention of the psychotherapist.

As a result of Covid-19 - an infectious disease caused by the SARS-CoV-2 coronavirus - the clinic’s activities were interrupted in March 17, 2020, with a gradual return on May of the same year, with cases selected by women’s age, serious male injuries or oncological procedures. The psychotherapist’s return to activities only took place as of July 2020, when the transfers were also progressively restarted. The psychotherapist was present in 238 cases, corresponding to 79.5% of the total of 299 performed by the clinic in 2021, and there was an increase in the number of embryo transfers performed in 2022; where 197 cases received psychological assistance (83.47% of the total of 236 embryo transfers). The data are expressed in Table 1 and Figure 1.

**Table 1:** Presence of the psychotherapist. (2019-2022 Procedures).

Procedure	2019	2020	2021	2022	2019-2022
	n/Intervention	n/Intervention	n/Intervention	n/Intervention	n/Intervention(%)
ICSI	238/161	179/132	299/238	284/64	1000/595(60%)
Oocyte cryopreservation	81/33	78/19	130/30	107/28	396/110(28%)
TE	169/30	116/27	232/52	236/197	753/306(41%)
Total	488/224	373/178	661/320	627/289	2149/1011(47%)

## DISCUSSION

“*Is there anyone who doesn’t get nervous in this place?*”

*“I feel like I’m in the delivery room, I wish my mom was here.”*

“*May our Lord protect and sustain you all!”*

Upon arriving at OSC of the assisted reproduction clinic, the patient initially faces the need to get undressed. Naked, she is “completely or partially *desnuda* (unclothed)”, “she allows her state of mind to be shown”. What initially was a contact, a relationship when the patient first enters the clinic having her first attendance with a doctor, turns to be different contacts, once, from now on, she will be assisted by a multidisciplinary team, made up of doctors, nurses, psychotherapists, nursing technicians, pharmacists, receptionists, maids and general service assistants. Nothing will stay out with the patients’ clothes. Their stories, experiences, subjectivities and, above all, their psychological reality, will come with them into the surgical center.

“*There are many emotions, they all come together!*”

(Patient*’*s speech addressed to a psychotherapist in the OSC environment)

We observe that anxieties, fears, insecurities, frustrations, fantasies, anguish and the feeling of helplessness, “all come together”. The emptiness of the womb that has not been occupied for a period, or for a long time, and facing this condition, in view of the procedures performed at the OSC, makes present, for some patients, the feeling and experience of helplessness. With the intention of supporting and welcoming the patient in what she is able to enunciate and reveal through her speech, we have observed that, sometimes, listening to the feeling of helplessness has been placed aside.

We see, therefore, a paradox: the one who is aiding, if not aware of the request for support, may be an agent of helplessness. Reis (2004) argues that “clinical experience is based on a request directed by someone who suffers for another, so that this one reveals the nature of the evil that torments him. [...] No one leaves unscathed from this process initiated by such request, once the protagonists affect each other in a delicate game of forces although in different places and roles” (p. 13).

The patient who comes to the ART clinic, especially to the OSC, desires and delivers her symptom - the difficulty to conceive -with the expectation of an offer of care and diagnose. However, the course of this process is a two-way path where both patient and team, participants of her experience, will be involved.

Ansermet (2003) states that “the clinic, at its core, is at the same time the practice of knowledge and a path of research that contributes to establishing knowledge from practice” (p. 7). It highlights some counterpoints, in our view, complementary, the interface between medicine and psychotherapy. According to this author, “while medicine is based on image, psychotherapy bets on speech. It is guided by what the person declares. Where medicine is a science of the body, as an object captured by the look, psychotherapy aims to be a clinic of the person supported by listening”. A non-passive listening, but an active listening, sensitive to what is said and not said by the other, which is able to withstand uncertainties. As the plastic artist Mana Bernardes (2019) says, “listening can be a useful womb that carries the sound and words of the other like a fetus”. We can consider, then, that analytical listening “becomes a fundamental instrument in the intervention of situations of helplessness and psychic pain” (Dockhorn *et al*., 2007) (p. 25).

By offering listening to support, assist and accompany patients in the OSC of the ART clinic, the psychotherapist can identify, from the speeches of the patients, the feeling of helplessness, whether in progress or precipitating before, during or after the IVF/ICSI procedure, cryopreservation of embryo transfer. When directing her speech to the psychotherapist or the team, the patient transfers contents and affections, making it possible to observe and identify the feeling of helplessness also experienced by the multidisciplinary team, especially when waiting for the results, number of aspirated oocytes and in the face of negative outcomes. Therefore, in the OSC environment, patients will often be faced with their weaknesses, faults, psychic realities and the uniqueness of their stories. As a result of this experience, they will be confronted with a reality that they would so much like to avoid, that is, using the ART technique to fulfill an unconsummated desire - pregnancy.

*“I came to Rio, to this clinic, by recommendation of someone I met in my city who lives here in Rio. This person has a colleague who underwent FIV here and it worked. He said he’d come with me and stay here with me, but at the last minute he backed out. I’m here alone, very anxious. My parents are doctors. I told them I would do the procedure. My mother supported me, my father did not. Now, I’m here without them and also without the support of the person who would be here with me. My father was always with me in the procedures I needed to do throughout my life; he stayed with me, he supported me. Now, I’m here without him knowing. Talking to you, I remembered my father, who supported me”.* (Patient’s speech addressed to a psychotherapist in the OSC environment).

The presence of helplessness can be observed from birth and in the first expressions of life of the human baby, “as a result of the incompleteness of the organism, its need to exchange with the world and its extreme dependence on the help of others”. (Oliveira *et al*., 2014). Helplessness, defined in the dictionary of the Portuguese language as the “lack of support, aid or protection; abandonment”, helplessness or the state of helplessness undeniably puts us in a relationship with the other, establishing bonds and social interactions.

In Freud, the notion of helplessness is present since the beginning of his work (Freud, 1977). We find the word helplessness in the text *Project for a Scientific Psychology* discovered 50 years after his death, where he deals with the “experience of satisfaction”. In this context, Freud talks about helplessness, explaining that

*The human organism is, at first, unable to promote this specific action. It is carried out by others’ help, when the attention of an experienced person deals with a childish state by discharge through change of path. This outlet thus acquires the important secondary function of communication, and the initial helplessness of human beings is the main source of all moral reasons*.

Freud (1977) also comments that “to say that helplessness is at the base of morality is to support how the figure of the other interferes from the beginning in the formation of the person” (p. 336).

Pereira (1999) highlights the presence of the feeling of helplessness since birth in the newborn. He points out that the theme of helplessness was placed at the beginning of Freud’s work “in very concrete terms of the newborn’s objective inability to satisfy, by its own strength, the requirements of its vital needs, to, later, be resumed and re-elaborated [...]” (p. 127). Following this, Resstel (2015) stated that “the baby needs to have the other to perform specific actions, mobilizing in the other a feeling of asking for help to satisfy his needs” (p. 93), which would lessen the tension, provide a sense of relief and avoid the state of helplessness. Therefore, when the mother or a person who takes the place of the baby caregiver or someone else is unable to observe, interpret and above all support their needs, the baby may fall into disrepair.

**Figure 2:**
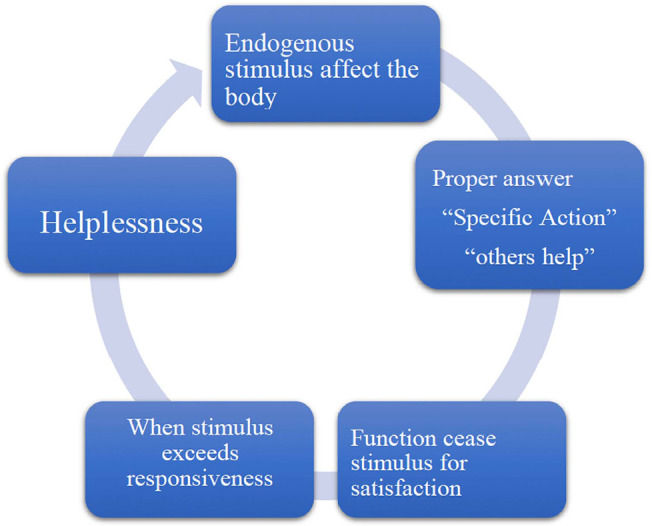
Patients’ tensions inside the OSC and Helplessness.

In this sense, in a similar way, we can consider that, when a patient enters the OSC to perform her procedure, endogenous and psychological stimuli may affect her organism; the expected “specific action” will be the support of their tensions and fantasies by “others ‘help”, that is, by a psychotherapist, a doctor and a multidisciplinary team, thus avoiding tension, frustration and the feeling of helplessness, as shown in Figure 2.

Also considering the context of the first days of a baby’s life, psychotherapist Ana Suy states that:

we come to life without a body, although we have an organism. we don’t know who we are, we do not recognize ourselves in the mirror [...]. We come to life so fragile that we depend on someone to adopt us to survive. [...] It is the desire of another that we exist that connects us to our flesh as an effect of the bet on that first other (or first others) who will love us (Suy, 2022) (p. 35).

Suy (2002) considers that “being welcomed into life with love is a question of life or death for human beings” and that “without human acceptance there is no chance for us, because someone’s love provides the foundation of life” (p. 24). When someone desires us, it involves us with life.

However, love and care, experienced in different ways in relationships with others, sometimes fail to accommodate the helplessness and anguish present in a given person. In this way, helplessness and anguish will be addressed by social relations, to the bonds established with others, to complete and deal with the experience of dissatisfaction caused by the lack of support felt by the subject in question.

It is worth mentioning Besset’s considerations, when she states that:

“if we speak of helplessness, we designate a state and when we speak of anguish, we refer to the affection of that person. Therefore, it is a primordial, first anguish, which is supposed at the base of the person’s birth, which would be about helplessness. Not an anguish-signal, which has a function, to prevent the I from the threat of a danger linked to the trauma” (Besset, 2002) (p. 212).

When the patient looks for the AR clinic to deal with something that she lacks and becomes distressed, resulting a state of helplessness, she does so in the expectation of feeling supported by the presence and clinical management of other types of knowledge. When the multidisciplinary team is aware of this demand, they will be able to welcome and listen to this patient in her uniqueness in the face of her desire - to have a child.

*“I get anxious because of the sedation”*

(Patient’s speech addressed to the anesthesiologist and the psychotherapist in the operating room).

“*Nobody likes to lose control. But, here we have no control It is very important to be welcomed here”.*

(Patient’s speech to the team, before being sedated. The team waits for a while, listens and supports the patient’s feelings before starting the procedure).

**Figure 3:**
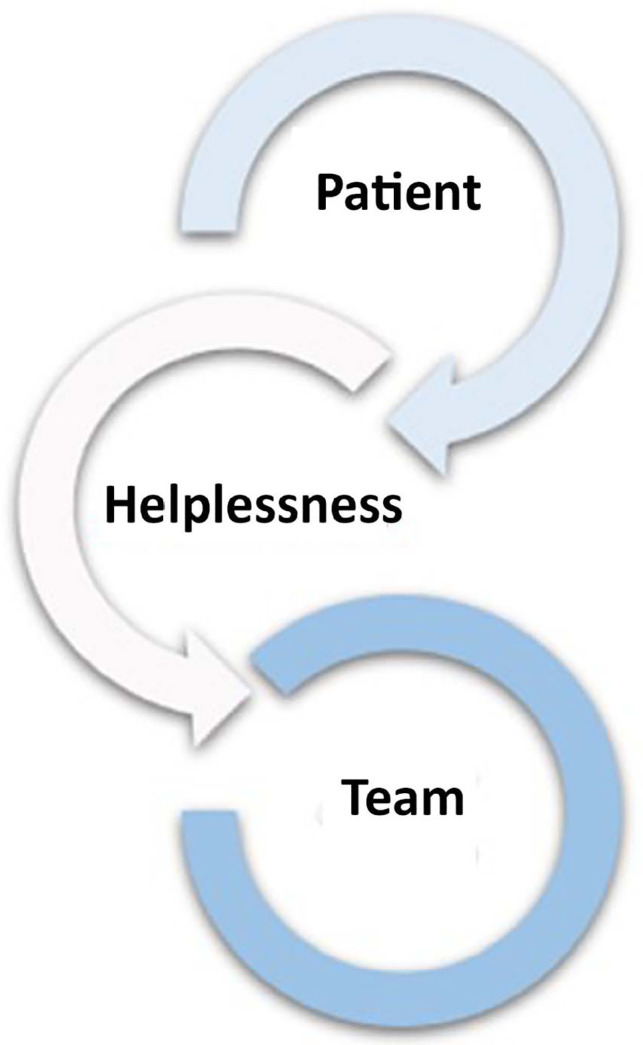
Helplessness deal with patients and the teams.

The patient expresses and explains in words, or in her behavior in general, her helplessness and addresses it to the team, which, therefore, to support her, will have to deal with her own helplessness, as shown in Figure 3.

The notion of helplessness gained greater prominence in Freud’s text *Inhibition, Symptom and Anxiety* (Freud, 1926). The psychological apparatus at this point in Freud’s work had already been reformulated in terms of id, ego and superego. In this text, Freud uses of the term helplessness, when he begins to investigate the origin of anguish; he notes that “the ego is reduced to a state of helplessness in the face of excessive tension due to need, as occurred in the situation of birth and that anguish is then generated “ (p. 165). Laplanche & Pontalis (1992) (p. 112) wrote that helplessness would be “the prototype of the situation that generates anguish”.

Dangerous situations of psychological helplessness, determinants of anguish, originating from the various stages of psychological development, may also arise in later situations throughout a person’s life. It will not be different with patients who look for an ART clinic to undergo procedures that will or will not allow the birth of a child. There will be uncertainties and lack of absolute guarantees, which both patients demand to obtain and hear from the team and will be confronted with their anxieties, anguish, helplessness and many other feelings. These can be experienced and felt in a unique way by patients, at each stage of the procedures, that is: in the application of medications; during the monitoring of follicular evolution; on the day of gamete aspiration; in response to monitoring the evolution of the embryo; on the day of embryo transfer; waiting for the result of the BHCG exam and waiting for the positive or negative response to a pregnancy. All these moments may be sources of feelings of helplessness for some patients and may even trigger psychopathological defenses.

*“I struggled a lot to be here at this moment. It’s all very exhausting. After a lot of waiting and trying, I had a natural pregnancy, but I lost it. It was a miscarriage, and it was just this year... Everything is on the surface, but... time passes, we get depressed, frustrated, recover and come back. Because there is a dream, because of age, because there was a loss and then I had to do the IVF to be able to do the PGT-A... Oh, I’m (sic) very tired of everything... especially the weather. I’m here for lack of options, but I found support, clarity and transparency... it’s a little lighter”.*

(Patient’s speech addressed to a psychotherapist in the OSC environment)

In his text *The Future of an Illusion,*Freud (1974a) states that

the terrifying impression of helplessness in childhood aroused the need for protection - for protection through love, which was provided by the father; the recognition that this helplessness lasts throughout life made it necessary to cling to the existence of a father, but this time a more powerful one. Thus, the benevolent government of a divine Providence mitigates our fear of the dangers of life (p. 43).

Therefore, once helplessness exists in children and adults, the search for a figure of a divine father in religions would ensure the subject’s protection for his state of helplessness. However, for Freud, religions are considered illusions, and religious ideas arose from the need to defend against the grandeur of Nature. Thus, the condition of helplessness would accompany the subject throughout his/her existence.

*“I never give up on what I want, I’m very persistent, but I let time pass too much. After I finished my master’s degree, I decided to get pregnant. I thought I could, but now I see that I have my age as an enemy. I never thought it would be so difficult. I will use donor semen. I already did a cycle that didn’t evolve. Due to my age, the ideal would be a donor egg, but I don’t want it. Take good care of my eggs! I believe in God and Science!! May Our Lady help us!”*

(Speaking of the patient addressing Science (team) and religion with her request for support in the face of her state of helplessness in the OSC).

In the text *Civilization and Discontents*, Freud (1974b) investigates and writes about the vicissitudes of the human being facing the social, the civilization. He points out that in order to live in society, human beings must make sacrifices, deprive themselves of pleasure and aggressiveness. By acting in this way, he would avoid losing the love of the other, the feeling of guilt, the risk of being abandoned, and he would not be abandoned by the divine Father. When referring to helplessness, he considers that it is inherent to the human condition and that the relationships established between human beings and civilizing forces can place the subject facing his social helplessness.

Birman (2000) summarizes very well the paths that Freud took in his texts when he mentions helplessness when he tells us that he “outlined the position of structural fragility of the subject, by relating this to his corporeality, to the threats of nature and to the horrors generated in the ambivalent relationships with others [...]” (p. 36). He points out that “the psychological record of helplessness is something original, marking human subjectivity forever and ever, in an indelible and indisputable way” (p. 37). Birman (2000) also considers that “abandonment would be what creates malaise in modernity” (p. 43).

Crossings of a time of social helplessness, from the Covid-19 situation, were present in the life of the multidisciplinary team and in the experience of patients, as we can see in the report below.

*“I’m going to transfer two fresh embryos. Because of my age I didn’t take the PGT-A. I really wanted to have twins or triplets. I lost my parents to Covid-19, my aunt and godmother, who is here with me, gives me the strength I need. I’m very happy and excited. I am hopeful that everything will be alright!”.* (The patient sought the clinic to perform the embryonic transfer shortly after the clinic’s recess period due to Covid-19. She reported to the psychotherapist her hope of getting pregnant and rebuilding her family, given the loss of her parents to Covid-19).

The idea of contemporary individual lives in a time of helplessness is present in Oliveira *et al*. (2014) as discussed by many other authors. The modus operandi of current relationships (see social networks) marked by greater exposure, lack of privacy and individualism, can imprint a state of helplessness on the subject, as the greater number of contacts or likes does not guarantee her a condition of support, in view of the weaknesses of the relationships established in these social ties. “The subject finds herself, on the one hand, with greater freedom for possibilities of being and contemplating the singularity of her desire and, on the other hand, she finds herself in a situation of helplessness in the face of the uncertainty and insecurity that such a state of affairs can cause” (Oliveira *et al*., 2014).

Psychotherapist Fabio Belo (2022) points out that it is important to understand the notion of helplessness beyond a biological dimension. He mentions that helplessness concerns the “ego”. He considers that “it is not before the world, civilization, social demands, that the subject is distressed, but, yes, before his desire”. He points out that helplessness is the instinctual attack on the “ego” - a feeling of anguish arising from an instinctual attack towards her, He emphasizes that helplessness is the instinctual attack on the “ego” - a feeling of anguish arising from an instinctual attack towards the “ego”. As the attack is no longer inherent to the baby’s psychophysiological surfaces, it will concern something that the subject locates as an attack on his “ego”, on his unconscious desire. It is the adult who gives meaning, welcomes the baby into his fantasy that he is helpless, passive. It is this passivity produced in this scene of meaning, in this libidinal scene, which comes back to all of us a while later as helplessness that we live, in fact, without having been there. A passivity that we do not experience in the self, but we experience it as a body, someone who was beginning to constitute himself as a subjectivity” (Belo, 2022).

**Figure 4:**
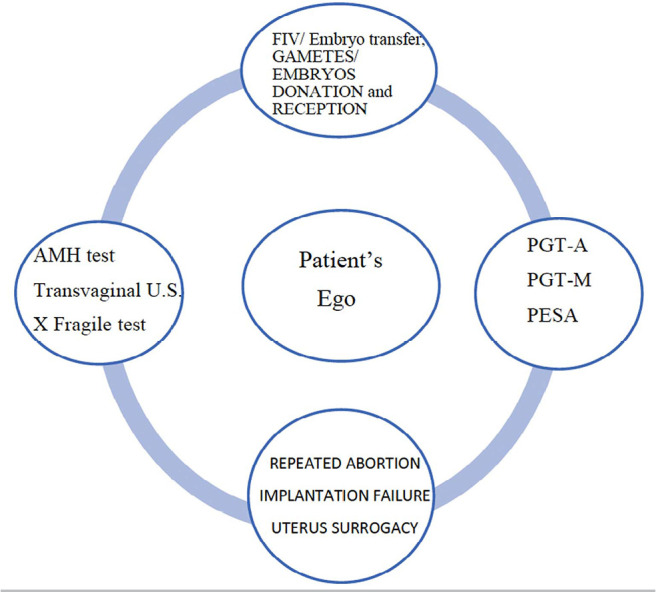
Would the procedure be understood as an attack on the patients’ ego?

Based on the above considerations, could medications, tests and procedures performed by patients in the AR clinic be understood as an “attack” on the patient’s “ego”? Would they cause the patient’s ego to become helpless? Since these procedures may or may not indicate paths to the condition of becoming pregnant, in which the uncertainty of previous results, sometimes, may produce anguish, would they put patients in front of their *ego-desire-helplessness? Worth the thought!* (Figure 4)

*“Hold my hand because I can’t handle it. Take care of me!”*

(Patient’s speech addressed to the psychotherapist in the OSC)

It will be due to what makes her helpless, in the face of his lack, that the patient will mobilize her desire, making choices and seeking destinations that can account for her existential helplessness. The ART clinic can be a destination chosen by patients to face their state of helplessness, experienced in the face of the impossibility of fulfilling their desire - to have a child.

We observed a counterpoint: if, on the one hand, clinic professionals as a whole need to place themselves in a place of continent (womb), in order to be able to accommodate the emotional and affective experiences of patients; on the other hand, they also need to be aware of and be supported in the face of their own helplessness. See the effects of the negative outcomes experienced in the team. Therefore, we believe that attention directed towards helplessness can produce a greater understanding of its effect on a person, on relationships and on the destiny that each one will try to give to their existential helplessness.

“Everyone has to cling to something to keep living” (Passos, 2022) (p. 116).

## CONCLUSIONS

From a psychoanalytical clinical practice, it was possible to identify and give way to listening to the feeling of helplessness experienced by patients and the team in the ASC of an Assisted Reproduction clinic.

The speech of the patients addressed to a professional or a multidisciplinary team, in the ASC environment, contains the statement of their feelings, loaded with conscious and unconscious contents that affect their psyche. In this context, the feeling of helplessness, expressed and stated in the speeches and behaviors of patients, as well as the team, can go unnoticed and sometimes, not receive the necessary care.

Attention directed towards helplessness can produce a greater understanding of its effect on subjects, on relationships and on the destiny that each one will try to give to their existential helplessness. Faced with the difficulty of getting pregnant, the helplessness of the self, in the AR technique can be a way to go.

The psychoanalytic intervention carried out in the outpatient surgical center of the assisted reproduction clinic can reflect and enable the understanding of “our daily helplessness”, both in patients and in the team, based on the sensitive listening of the subjects involved in this scenario.

## References

[r1] Ansermet F. (2003). Clínica da origem: a criança entre a medicina e a
psicanálise.

[r2] Belo F. Desamparo.

[r3] Bernardes M. (2019). Ritos do nascer ao parir.

[r4] Besset VL. (2002). Angústia e desamparo. Rev Mal-Estar Subj.

[r5] Birman J. (2000). Mal-estar na atualidade: a psicanálise e as novas formas de
subjetivação.

[r6] Braverman AM, Davoudian T, Levin IK, Bocage A, Wodoslawsky S. (2024). Depression, anxiety, quality of life, and infertility: a global
lens on the last decade of research. Fertil Steril.

[r7] Brazil, Ministério da Saúde, ANVISA - Agência Nacional de Vigilância
Sanitária (2022). Reprodução Humana Assistida. Norma atualiza boas
práticas em células, tecidos e embriões
humanos.

[r8] Dockhorn CNBF, Macedo MMK, Werlang BSG. (2007). Desamparo e dor psíquica na escuta da
psicanálise. Barbarói.

[r9] Freud S. (1926). Inibições, sintomas e angustia.

[r10] Freud S. (1974a). O futuro de uma ilusão.

[r11] Freud S. (1974b). O mal-estar na civilização.

[r12] Freud S. (1977). Projeto para uma psicologia científica. Vol.
I.

[r13] Golombok S. (1992). Psychological functioning in infertility patients. Hum Reprod.

[r14] Gusmão MCG, Teixeira LM, Mancebo ACA, Souza MM, Antunes RA, Souza MDCB. (2020). Psychological Intervention in the Oocyte Pick-up Room and
Recovery Room in Assisted Reproduction: new listening
accounts. JBRA Assist Reprod.

[r15] Kislanov SA. (2002). Em busca de um rosto: uma clínica psicanalítica com
pacientes submetidos a cirurgias reconstrutoras da face.

[r16] Laplanche J, Pontalis J. (1992). Vocabulário de Psicanálise.

[r17] Oliveira AAA, Resstel CCFP, Justo JF. (2014). Desemparo psíquico na contemporaneidade. Rev Psicol UNESP.

[r18] Passos CF, Neves AS, Menezes LS. (2018). Prolegômenos do desamparo na
psicanálise. Rev Latinoam Psicopatol Fundam.

[r19] Passos V. (2022). A filha primitiva.

[r20] Pereira MEC. (1999). Pânico e desamparo: um estudo psicanalítico.

[r21] Reis ES. (2004). De corpos e afetos: transferências e clínica
psicanalítica.

[r22] Resstel CCFP. (2015). Desamparo psíquico nos filhos de dekasseguis no retorno ao
Brasil.

[r23] Rooney KL, Domar AD. (2018). The relationship between stress and infertility. Dialogues Clin Neurosci.

[r24] Suy A. (2022). A gente mira no amor e acerta na solidão.

